# Dietary Fiber Influences Bacterial Community Assembly Processes in the Gut Microbiota of Durco × Bamei Crossbred Pig

**DOI:** 10.3389/fmicb.2021.688554

**Published:** 2021-12-08

**Authors:** Xianjiang Tang, Liangzhi Zhang, Chao Fan, Lei Wang, Haibo Fu, Shi’en Ren, Wenjuan Shen, Shangang Jia, Guofang Wu, Yanming Zhang

**Affiliations:** ^1^Key Laboratory of Adaptation and Evolution of Plateau Biota, Northwest Institute of Plateau Biology, Chinese Academy of Sciences, Xining, China; ^2^Plateau Livestock Genetic Resources Protection and Innovative Utilization Key Laboratory of Qinghai Province, Qinghai Academy of Animal and Veterinary Medicine, Qinghai University, Xining, China; ^3^Qinghai Provincial Key Laboratory of Animal Ecological Genomics, Xining, China; ^4^University of Chinese Academy of Sciences, Beijing, China; ^5^College of Grassland Science and Technology, China Agricultural University, Beijing, China

**Keywords:** dietary fiber, gut bacteria, spatial heterogeneity, community assembly, Durco × Bamei crossbred pig

## Abstract

Several studies have shown that dietary fiber can significantly alter the composition and structure of the gut bacterial community in humans and mammals. However, few researches have been conducted on the dynamics of the bacterial community assembly across different graded levels of dietary fiber in different gut regions. To address this, 24 Durco × Bamei crossbred pigs were randomly assigned to four experimental chows comprising graded levels of dietary fiber. Results showed that the α-and β-diversity of the bacterial community was significantly different between the cecum and the jejunum. Adding fiber to the chow significantly increased the α-diversity of the bacterial community in the jejunum and cecum, while the β-diversity decreased. The complexity of the bacterial network increased with the increase of dietary fiber in jejunal content samples, while it decreased in cecal content samples. Furthermore, we found that stochastic processes governed the bacterial community assembly of low and medium dietary fiber groups of jejunal content samples, while deterministic processes dominated the high fiber group. In addition, deterministic processes dominated all cecal content samples. Taken together, the variation of gut community composition and structure in response to dietary fiber was distinct in different gut regions, and the dynamics of bacterial community assembly across the graded levels of dietary fiber in different gut regions was also distinct. These findings enhanced our knowledge on the bacterial community assembly processes in gut ecosystems of livestock.

## Introduction

Dietary fiber is the major substrate for intestinal bacteria in livestock (Chen et al., 2014; Mao et al., 2015; Zhang et al., 2016). Previous studies have shown that dietary fiber can significantly alter the composition and structure of the gut microbes of host animals, resulting in alterations in their functions and metabolites (De Filippo et al., 2010; Schnorr et al., 2014; Heinritz et al., 2016; Zhang et al., 2016; Chen et al., 2017; Sheflin et al., 2017; Zhao et al., 2018; Li G. et al., 2020; Morrison et al., 2020; Pu et al., 2020). Markedly, increasing the proportion of fiber in the swine diet enhances the α-diversity and β-diversity indices of the gut microbiota of the host animals (Heinritz et al., 2016; Pu et al., 2020). Adding a fiber source to the chow of piglets significantly increased the relative abundance of gut bacteria phyla, such as *Firmicutes*, *Actinobacteria*, and *Fibrobacteres*, which can ferment dietary fiber and produce large amounts of short-chain fatty acids (SCFAs) (Zhao et al., 2018). Most of these studies have focused on the effects of dietary fiber on the composition, structure, and function of gut microbiota. However, whether dietary fiber could affect the microbial community assembly processes in the gut ecosystem of these livestock is still poorly understood.

The mammalian gastrointestinal tract is a long and connected lumen with a distinct composition and structure of epithelial cells in different regions (Brunsgaard, 1997). Such variation could influence the host secretions, luminal PH and gut motility, then lead to the significant distinction of on composition and structure of gut microbiota among regions of gastrointestinal tract (Mao et al., 2015). The studies on Laiwu pigs, dairy cattle, goats, roe deer (*Capreolus pygargus*) and pikas (*Ochotona curzoniae*) all reveal spatial heterogeneity of the bacterial community in the gastrointestinal tract (Li et al., 2014, 2017; Mao et al., 2015; Yang et al., 2016; Wang et al., 2019). Additionally, as the function of the host gut in digesting food resources is distinct in different regions of the gastrointestinal tract, resulting in different nutrient supplies for microbiotas which may also contribute to the spatial heterogeneity of the bacterial community. Therefore, the taxonomic diversity, community composition, and assembly of bacteria in different gut regions in responding to the variation of dietary fiber may be different.

Over the past few decades, the factors responsible for species diversity, microbial community stability and assembly have been the central topic in ecological research (Hutchinson, 1959; Zhou and Ning, 2017). Ecologists have postulated niche-based and neutral-based theories to solve these questions (Chave, 2004; Rosindell et al., 2011). The neutral theory hypothesizes that all species are ecologically functionally equivalent and that the variation in species abundance is controlled by birth, colonization, death, extinction, or speciation, which are referred to as stochastic processes (Hengeveld, 2002; Chave, 2004; Etienne and Olff, 2004; Hubbell, 2010). Contrarily, the niche-based theory emphasizes that environmental factors (e.g., temperature, pH, and moisture) and biotic factors, such as species traits and interspecies interactions (e.g., competition and predation), govern the community structure and are referred to as deterministic processes (Peter, 2000; Fargione et al., 2003). Recently, community assembly has been accepted to be simultaneously governed by deterministic and stochastic processes (Ofiteru et al., 2010; Chase et al., 2011; Stegen et al., 2012). Numerous studies have focused on the gut community assembly of animals, including lobsters, fishes, pikas, and humans, and have explored the influence of host developmental stage and environmental factors, such as habitat, elevation, and temperature, on the assembly processes of bacterial communities (Martínez et al., 2015; Yan et al., 2016; Li et al., 2019; Holt et al., 2020). Studies on fishes suggest that gut bacterial community assembly processes vary across the host development stages. The community assembly of individuals in the larval stage is dominated by the deterministic processes, while it is dominated by stochastic processes in the adult stage (Yan et al., 2016), indicating that a variation in host physiological features influences the gut bacterial community assembly. The research regarding pikas and murine suggests that the distinct living environment and food resources also influence the community assembly processes of gut bacteria (Carmody et al., 2015; Li et al., 2019). However, knowledge of the effect of graded dietary fiber on the host gut bacterial community assembly in different gut regions is limited.

The Bamei pig (*Sus scrofa*) is a common livestock species in the Tibetan Plateau (Zhou et al., 2016). They have a greater ability to digest high fiber diets than wild pigs, as their crude fiber digestibility is significantly higher (52.08%) than that of wild pigs (45.72%) (Gun et al., 2007). Dietary fiber is fermented by gut bacteria, which produce SCFAs that are needed for the growth of beneficial bacteria in the gut (Jha and Leterme, 2012). For this study, female Bamei and male Duroc pigs were crossed to form Duroc × Bamei hybrids that possessed superior fiber digestibility and growth rate compared to their parents (Gun et al., 2007; Zhou et al., 2016). These unique features made them suitable for studying the gut microbial community assembly using different dietary fiber levels. Here, we used the jejunal and cecal content of Duroc × Bamei crossbred pigs from four dietary fiber groups to investigate the dynamics and community assembly of gut bacteria. We aimed to evaluate the following: (1) whether the spatial heterogeneity of gut regions is a significant factor in shaping the bacterial community, because the different gut regions are usually associated with distinct composition and structure of epithelial cells which may result in a distinct microenvironment, (2) whether the variation in the composition and structure of the gut bacterial community in response to dietary fiber was distinct in different gut regions, and (3) whether the response of bacterial community assembly in different gut regions to dietary fiber was distinct. We hypothesized that the ratios of deterministic processes to stochastic processes across the four dietary groups varied in different gut regions because of the distinct microenvironments.

## Materials and Methods

### Ethics Statement

All experimental procedures were performed according to the Guide for Animal Care and Use of Laboratory Animals in the Institutional Animal Care and Use Committee of QingHai University. The experimental protocol was approved by the Department of Animal Ethics Committee of QingHai University (Approval number: NQH2019102).

### Animals

The experimental animals were hybrid offspring of male Duroc (*Sus scrofa*) and female Bamei pigs. Twenty-four (12 male and 12 female) 3-month-old pigs with average initial body weight of approximately 25.5 kg were obtained from the Qinghai Bamei pig breeding farm. Pigs were individually housed in a pen and had *ad libitum* access to water and the treatment diets.

### Experimental Design and Samples Collection

The experimental animals were randomly assigned into four treatment groups (Control, Groups I, II, and III; 6 pigs per group). Three males and three females were assigned to a group. Pigs in the control group were fed basic chow, while the chow of the pigs in groups I, II, and III were supplemented with 10, 17, and 24% silage, respectively. The ingredient composition and nutrient content of the basic diet is shown in Supplementary Table 1, and the nutrient and energy content of the silage were shown in Supplementary Table 2. None of the pigs received antibiotics or medicine before and during the experiment. After 90 days of the experimental period, the pigs were slaughtered, and the fresh intestinal contents in the jejunum and cecum were collected immediately and stored at −80°C.

### Intestinal Microbiota Sequencing

Fecal DNA was extracted from samples of the cecal and jejunal contents using a QIAamp DNA Stool Mini Kit (Qiagen 51504) according to the manufacturer’s instructions. DNA purification was performed using QIAamp Mini Spin columns following the standard protocol. The DNA concentration was determined using a NanoDrop ND-1000 (Thermo Fisher Scientific, Waltham, Massachusetts, United States). The 16S rRNA genes of 16S V4-V3 were amplified using specific primers (341F: 5′-CCTAYGGGRBGCASCAG-3′ and 806R 5′-GGACTACNNGGGTATCTAAT-3′) with the barcode. All PCRs were carried out with 15 μL of Phusion^®^ High-Fidelity PCR Master Mix (New England Biolabs). The PCR products were detected using 2% agarose electrophoresis gel. Thereafter, the PCR product was purified using a Qiagen Gel Extraction Kit (Qiagen, Germany). Sequence libraries were generated using a TruSeq^®^ DNA PCR-free sample preparation kit (Illumina, United States) following the manufacturer’s recommendations, and index codes were added. The library quality was assessed on a Qubit@ 2.0 Fluorometer (Thermo Fisher Scientific) and Agilent Bioanalyzer 2100 system. Finally, the library was sequenced on an Illumina NovaSeq platform, and 250 bp paired-end reads were generated. The raw reads were deposited into the NCBI SRA database with accession number PRJNA685300.

### Bioinformatics and Data Analysis

The raw sequences were normalized, filtered, and processed using QIIME Pipeline-Version 1.9.1^1^ (Caporaso et al., 2010). Sequences with average quality scores of less than 19 were removed. Chimeras were identified and removed using VSEARCH^2^ (Rognes et al., 2016), and the retained sequences were effective tags for downstream analyses. All effective tags were clustered into operational taxonomic units (OTUs) using Uparse v7.0.1001^3^ at 97% identity (Edgar, 2013). We chose the highest frequency sequences in each OTU as representative OTUs for further annotation. For each representative sequence, the Silva database^4^ (Quast et al., 2013) was used to annotate taxonomic information with a standard threshold of 80%, based on the Mothur algorithm (Wang et al., 2007). Taxonomic profiles were evaluated at the kingdom, phylum, and genus levels. Thereafter, the mitochondrion and chloroplast OTUs were removed before further analysis.

Statistical analyses were performed, and graphs were plotted using the R packages “vegan,” “phyloseq,” “picante,” and “NST” in R (v3.5.3^5^; R Foundation for Statistical Computing, Vienna, Austria) and SPSS v20.0. The Kruskal–Wallis test was used to assess the differences in the relative abundance of bacterial phyla and genera in the different dietary groups. The Wilcoxon test was used to determine the distinct diversity indices of the bacterial community of the cecal and jejunal content samples. Multiple-response permutation procedure (MRPP) and permutational multivariate analysis of variance (PERMANOVA) were used to determine the effect of dietary fiber on the composition of the bacterial communities (Yan et al., 2016). Principal-coordinate analyses (PCoA) based on Bray-Curtis and Jaccard distances were conducted to determine the effect of dietary fiber on the overall patterns of microbial communities. A null model analysis based on permutational analysis of multivariate dispersion (PERMDISP) was performed to find out whether the observed β-diversity values (Bray-Curtis and Jaccard distance) were significantly different from the null expectation (Chase et al., 2011). The mean nearest taxon distance (MNTD) and nearest taxon index (NTI) were used to determine the phylogenetic diversity of bacterial communities (Webb et al., 2002). An NTI value > + 2 or < −2 in one sample or a mean NTI value > 0 or < 0 in a community indicated significant phylogenetic clustering or phylogenetic overdispersion (Stegen et al., 2012). βMNTD measures MNTD between all pairs of OTUs distributed in two different samples, and βNTI measures the deviation of observed βMNTD from the mean of the null distribution. A βNTI value > + 2 or < −2 between one pair of samples or a mean βNTI value > 0 or < 0 of all sample pairs indicated a significant predominance of deterministic processes, otherwise the community was dominated by stochastic processes (Stegen et al., 2012). To quantify the contribution of each of the five ecological processes (homogenous selection, variable selection, homogenous dispersal, dispersal limitation, and drift) to community turnover, we calculated the weighted beta nearest taxon index (βNTI) and Bray-Curtis-based Raup-Crick (RC_bray_) according to the analytical framework of Stegen et al. (2012, 2013). A βNTI value <−2 indicated that the community turnover was determined by homogeneous selection, while a value >+2 indicated that the community turnover was determined by variable selection (Vellend, 2010; Martínez et al., 2015). An absolute βNTI value < + 2 and RC_bray_ value > + 0.95 between a pair of samples indicated that the community turnover was governed by dispersal limitation, while an RC_bray_ value <−0.95 indicated that the community turnover was governed by homogenizing dispersal processes (Stegen et al., 2013; Martínez et al., 2015). An absolute βNTI value between −2 and +2 and RC_bray_ value between −0.95 and + 0.95 indicated that the community turnover was governed by drift processes (Stegen et al., 2015). An NST value > 50% indicated that stochastic processes dominated the community assembly, otherwise the deterministic processes dominated (Ning et al., 2019).

## Results

### Effect of Dietary Fiber on Bacterial Taxa Composition in the Jejunum and Cecum

From the 48 samples of the jejunum (*N* = 24) and cecum (*N* = 24) obtained from the Durco × Bamei crossbred pigs, we obtained 3,958,902 high-quality gene sequences of the bacterial 16S rRNA. These sequences clustered into 3,457 and 2,119 OTUs (UPARSE, 97% cutoff) in the jejunal and cecal content samples, respectively. *Firmicutes* were the most dominant bacterial phyla both in the jejunum (80.76%) and cecum (84.76%), followed by *Proteobacteria* (10.29%) and *Actinobacteria* (7.58%) in the jejunum, and *Bacteroidetes* (9.97%) and *Actinobacteria* (1.54%) in the cecum (Supplementary Figure 1A). The most dominant genera in jejunum were unidentified *Clostridiales* (17.59%), *Terrisporobacter* (14.23%), *Lactobacillus* (12.34%), *Streptococcus* (10.40%), and *Romboutsia* (8.79%), while in the cecum, they were unidentified *Ruminococcaceae* (14.25%), *Terrisporobacter* (13.60), unidentified *Clostridiales* (13.17%), *Turicibacter* (5.51%), and *Romboutsia* (3.77%) (Supplementary Figure 1B).

**FIGURE 1:**
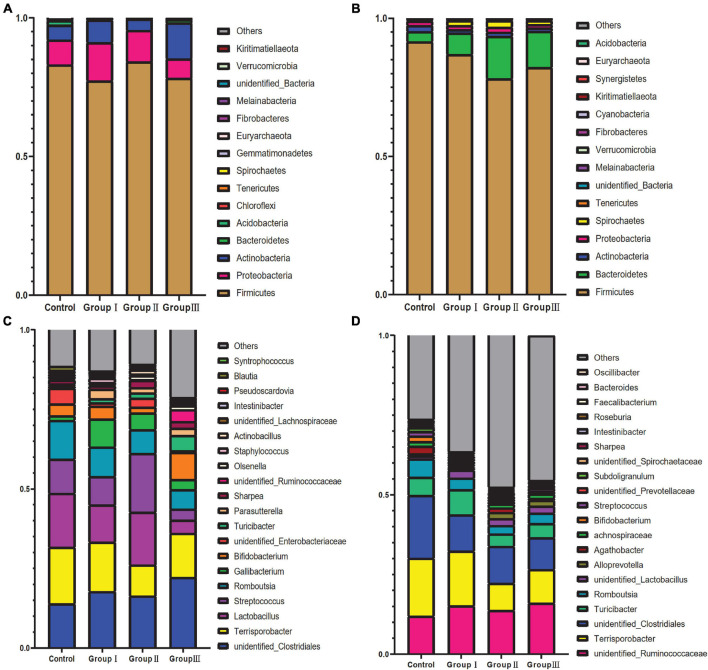
Taxonomic composition of gut bacteria of four dietary fiber groups from jejunal and cecal content samples. The relative abundance of the 15 most abundant bacterial phyla from jejunal content samples **(A)** and cecal content samples **(B)**. The relative abundance of the 20 most abundant bacterial genera from the jejunal content samples **(C)** and cecal content samples **(D)**.

There were significant differences on the relative abundance of dominated bacteria phyla and genera among four dietary fiber groups in the jejunum and cecum (Figure 1). *Firmicutes*, *Proteobacteria*, and *Actinobacteria* were the top three abundant bacterial phyla in the jejunum of all four dietary groups (Figure 1A). Among the top fifteen abundant bacterial phyla in the jejunum, we only detected the relative abundance of *Spirochaetes* to be increased in group III compared to the control group (Kruskal–Wallis test, χ^2^ = −11.250, *P* = 0.003). *Firmicutes*, *Bacteroidetes*, and *Actinobacteria* were the top three abundant bacterial phylum in the control group cecum, while *Spirochaetes* was the third most abundant bacterial phylum in the three treatment groups (Figure 1B). Kruskal–Wallis tests showed that of the top fifteenth abundant bacterial phyla in the cecum, the relative abundance of *Bacteroidetes* (Kruskal–Wallis tests: χ^2^ = 10.007, *P* = 0.019), *Tenericutes* (Kruskal–Wallis test: χ^2^ = 11.198, *P* = 0.011), and *Fibrobacteres* (Kruskal–Wallis test: χ^2^ = 11.380, *P* = 0.010) increased with the increase in dietary fiber, while the relative abundances of *Firmicutes* (Kruskal–Wallis test: χ^2^ = 8.140, *P* = 0.043) and *Cyanobacteria* (Kruskal–Wallis test: χ^2^ = 10.241, *P* = 0.017) decreased (Figure 1B). The most abundant bacterial genera in the jejunum varied among the four groups (Figure 1C). The bacterial genera with the greatest richness in the control group, group I, and group II were *Terrisporobacter*, *Lactobacillus*, unidentified *Clostridiales*, *Romboutsia*, and *Streptococcus*, whereas in group III, they changed to unidentified *Clostridiales*, *Terrisporobacter*, *Bifidobacterium*, *Romboutsia*, and *Turicibacter* (Figure 1C). Of the top 20 abundant bacterial genus in jejunum, the relative abundance of *Turicibacter* significantly increased with the increase in dietary fiber (Kruskal–Wallis test: χ^2^ = 17.407, *P* = 0.001), while the relative abundance of *Lactobacillus* decreased in group III, compared with control group (Kruskal–Wallis test: χ^2^ = 9.073, *P* = 0.028). The relative abundance of *Olsenella* only increased in group II (Kruskal–Wallis test: χ^2^ = −11.833, *P* = 0.004) and *Syntrophococcus* increased in group I (Kruskal–Wallis test: χ^2^ = −8.083, *P* = 0.048) and group II (Kruskal–Wallis test: χ^2^ = −12.333, *P* = 0.003) compared with control group (Figure 1C). The most abundant bacterial genera in all four dietary groups in the cecum were unidentified *Clostridiales*, *Terrisporobacter*, unidentified *Ruminococcaceae*, *Turicibacter*, and *Romboutsia* (Figure 1D). The relative abundance of unidentified *Prevotellaceae* (Kruskal–Wallis test: χ^2^ = 9.720, *P* = 0.021) and *Oscillibacter* (Kruskal–Wallis test: χ^2^ = 20.342, *P* = 0.000) increased with the increase in dietary fiber, while the richness of *Romboutsia* (Kruskal–Wallis test: χ^2^ = 13.767, *P* = 0.003), *Intestinibacter* (Kruskal–Wallis test: χ^2^ = 15.315, *P* = 0.002) and *Faecalibacterium* (Kruskal–Wallis test: χ^2^ = 12.328, *P* = 0.006) decreased with the increase in dietary fiber (Figure 1D).

### Effect of Dietary Fiber on the Diversity of Bacterial Communities in Jejunum and Cecum

**FIGURE 2:**
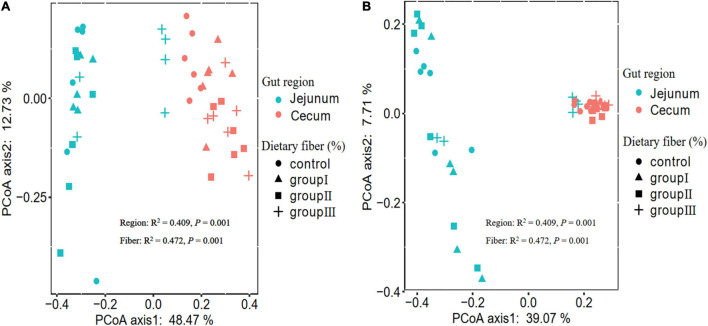
PCoA plots showing the different gut microbiota among the four dietary fiber groups between the jejunum and cecum based on Bray-Curtis **(A)** and Jaccard **(B)** distances. The significant effect of dietary fiber and gut regions on microbial β-diversity was detected using PERMANOVA.

Overall, the results of the two-way ANOVA showed that both gut location and dietary fiber significantly affected the Shannon, Simpson, and phylogenetic α-diversity of bacterial communities (Supplementary Table 3). The Shannon (Wilcoxon, *P* < 0.000), Simpson (Wilcoxon, *P* < 0.000), and phylogenetic α-diversity indices (Wilcoxon, *P* = 0.001) of the jejunum were lower than those of the cecum (Supplementary Figures 1C–E), indicating that the gut regions could alter the diversity of the host gut bacteria. The dissimilarity test based on Bray-Curtis and Jaccard distances indicated that the gut microbiota differed significantly between the jejunum and cecum (Table 1). The PCoA ordination calculated using the Bray-Curtis and Jaccard distances also showed that bacteria in jejunum was obviously separate from that in cecum (Figures 2A,B), although Group III with a high content of dietary fiber was clustered with cecum bacterial communities. This indicate that the bacterial taxa in the two gut locations were markedly different in the experimental pigs.

**TABLE 1:** Bray-Curtis and Jaccard distance-based dissimilarity tests showing differences in gut microbiota between the cecum and jejunum.

		Bray-Curtis				Jaccard			
		MRPP		PERMANOVA		MRPP		PERMANOVA	
		*Delta*	*P*	*F*	*P*	*Delta*	*P*	*F*	*P*
Gut region	Cecum vs. Jejunum	0.417	**0.001**	30.076	**0.001**	0.571	**0.001**	31.795	**0.001**
	Control vs. Group I	0.480	0.465	1.004	0.435	0.632	0.450	1.002	0.454
Jejunum	Control vs. Group II	0.489	0.400	1.061	0.342	0.639	0.520	1.084	0.325
	Control vs. Group III	0.483	**0.005**	2.985	**0.006**	0.630	**0.007**	2.968	**0.007**
	Control vs. Group I	0.297	**0.011**	3.046	**0.015**	0.453	**0.011**	3.175	**0.013**
Cecum	Control vs. Group II	0.306	**0.003**	5.377	**0.005**	0.466	**0.006**	5.635	**0.003**
	Control vs. Group III	0.302	**0.004**	5.154	**0.003**	0.460	**0.002**	5.286	**0.003**

*MRPP, multiple-response permutation procedure; PERMANOVA, permutational multivariate analysis of variance; Significant P-values < 0.05 (in bold).*

**FIGURE 3:**
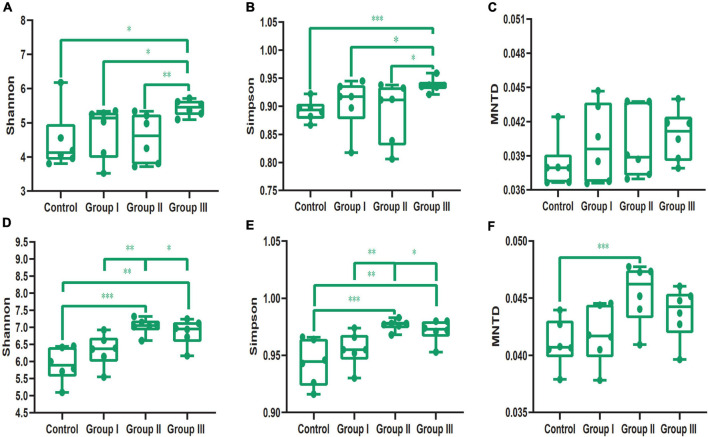
The diversity index comparisons of the bacterial communities from jejunal and cecal content samples. The Shannon’s **(A)**, Simpson’s **(B)** and phylogenetic α-diversity **(C)** indices of the four dietary fiber groups from jejunal content samples. The Shannon’s **(D)**, Simpson’s **(E)** and phylogenetic α-diversity **(F)** indices of the four dietary fiber groups from cecal content samples. Differences were assessed by the Wilcoxon test and are denoted as following: *P < 0.05; **P < 0.01; ***P < 0.001.

**FIGURE 4:**
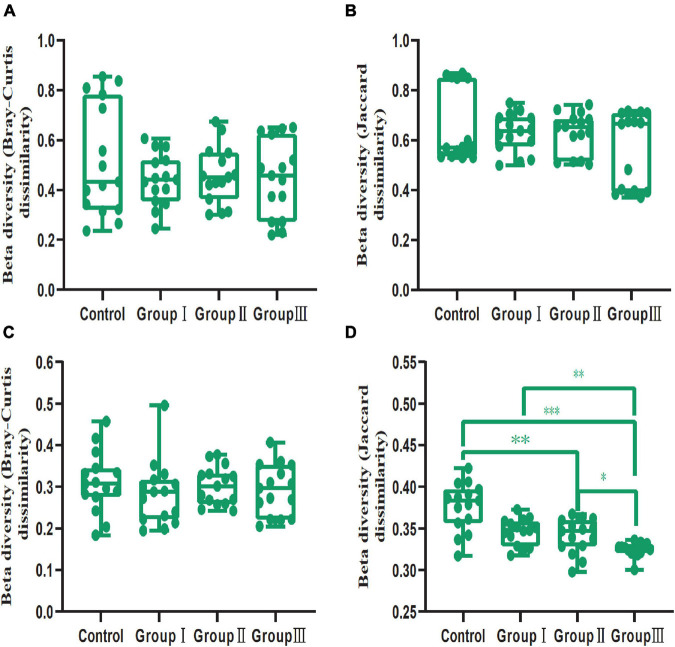
Comparison of β-diversity (Bray-Curtis and Jaccard dissimilarities within each dietary fiber groups) values between the four dietary fiber groups in the jejunum **(A,B)** and cecum **(C,D)**. Differences were assessed using the Wilcoxon test and are denoted as follows: *P < 0.05; **P < 0.01; ***P < 0.001.

In addition, our results showed that dietary fiber also could influence the diversity of the gut bacterial community (Figure 3). Both the Shannon (Wilcoxon, Group III vs. Control, *P* = 0.015; Group III vs. Group I, *P* = 0.040; Group III vs. Group II, *P* = 0.009) and Simpson (Wilcoxon, Group III vs. Control, *P* = 0.001; Group III vs. Group I, *P* = 0.043; Group III vs. Group II, *P* = 0.009) indices of group III were significantly higher than those of the control group, group I and group II in jejunum (Figures 3A,B). While in the cecum, the Shannon (Wilcoxon, Control vs. Group II, *P* < 0.001; Control vs. Group III, *P* = 0.002) and Simpson (Wilcoxon, Control vs. Group II, *P* < 0.001; Control vs. Group III, *P* = 0.001) diversity of group II and group III were significantly higher than that of the control group (Figures 3D,E); indicating that more dietary fiber is needed to determine the gut bacterial community in the jejunum than in the cecum. Additionally, the phylogenetic α-diversity index of cecum in group II was significantly higher than that of the control group (Wilcoxon, *P* = 0.008) (Figure 3F). The dissimilarity test based on the Bray-Curtis and Jaccard distances indicated that dietary fiber was an important factor in shaping β-diversity of the gut microbial community (Figure 2). In contrast to α-diversity, the β-diversity of the bacterial community in the cecum based on Jaccard distances was only significantly decreased in groups II and III compared with that of the control group (Wilcoxon, Control vs. Group II, *P* = 0.001; Control vs. Group III, *P* < 0.001) (Figure 4). In addition, we compared the pairwise differences of gut microbiota from our experimental pigs between the control group and treatment groups based on Bray-Curtis and Jaccard distances (Table 1). Bacterial community composition and structure in the cecum were different between the control group and the three treatment groups. However, only the control group and the highest dietary fiber group (group III) were significantly different in jejunum (Table 1).

### Effect of Dietary Fiber on Bacterial Networks

Network analyses based on the 20 most abundant genera showed that the bacterial taxa from cecal content samples were more complex than in the jejunum, as cecal bacteria possessed more links (Supplementary Figure 2). Generally, there were more positive correlations in each network, but the proportions of positive edges were higher in the network of cecal contents than those in the network of jejunal contents (Supplementary Figure 2). In the network of jejunal contents, the bacterial genera that possessed the greatest number of edges were *Terrisporobacter*, unidentified *Ruminococcaceae*, *Lactobacillus*, *Romboutsia*, *Streptococcus*, *Gallibacterium*, unidentified *Enterobacteriaceae*, and *Pasteurella*. Whereas, in the network of cecal contents, the bacterial genera with the greatest number of edges were unidentified *Clostridiales*, *Terrisporobacter*, *Romboutsia*, and *Streptococcus*.

**FIGURE 5:**
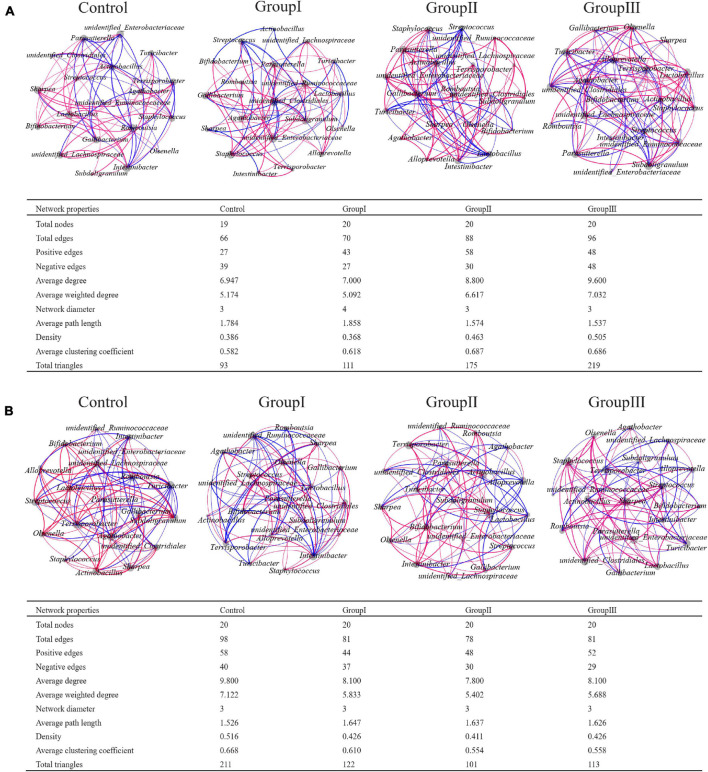
Co-occurrence networks of the 20 most abundant genera from the four dietary fiber groups in jejunal **(A)** and cecal **(B)** contents. Each line (blue: negative; red: positive) represents a Spearman’s correlation greater than +0.5 or lower than −0.5.

The total number of edges of the 20 most abundant genera from jejunal content samples increased as the dietary fiber increased, and the proportions of positive edges also increased in the high dietary fiber group (Figure 5A). In the control group, the bacterial genera that possessed the most links with other genera were *Terrisporobacter*, *Lactobacillus*, and *Romboutsia*, whereas in group I they were unidentified *Clostridiales*, unidentified *Ruminococcaceae* and *Lactobacillus* (Figure 5A); in group II, they were *Lactobacillus*, *Romboutsia*, and *Gallibacterium*; and in group III, they were unidentified *Clostridiales*, *Terrisporobacter*, and unidentified *Ruminococcaceae* (Figure 5A). In contrast to the jejunum, the total number of edges in the networks of the 20 most abundant genera from the cecal content samples decreased as the dietary fiber increased, while the proportions of positive edges increased (Figure 5B). The bacterial genera that possessed the most links with other genera are as follows: *Terrisporobacter*, unidentified *Clostridiales*, *Lactobacillus* and *Turicibacter* in the control group; unidentified *Ruminococcaceae*, unidentified *Clostridiales*, and *Terrisporobacter* in group I; *Terrisporobacter*, unidentified *Clostridiales*, *Streptococcus*, and *Bifidobacterium* in group II; and unidentified *Clostridiales*, *Terrisporobacter*, *Lactobacillus*, and *Turicibacter* in group III (Figure 5B).

### Effect of Dietary Fiber on the Assembly Processes of the Bacterial Community in the Jejunum and Cecum

**FIGURE 6:**
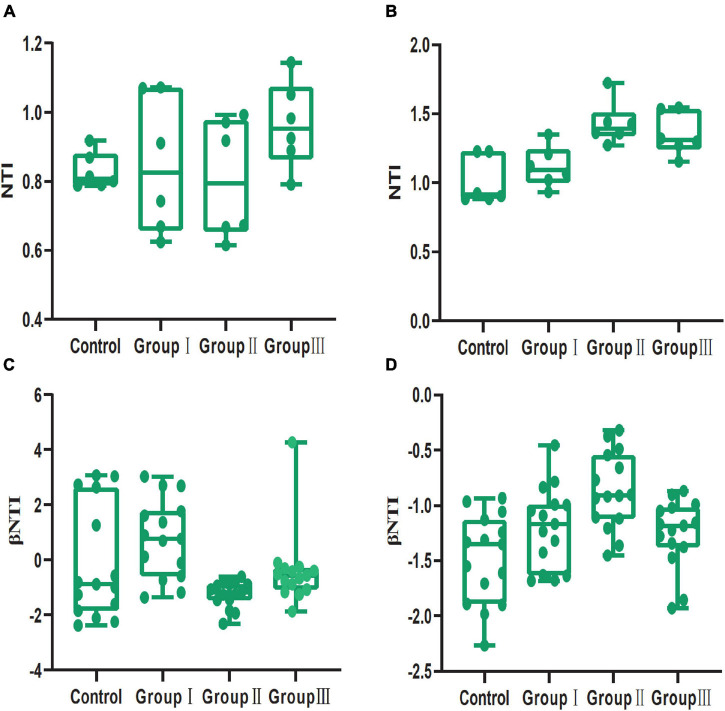
Box plot of NTI and βNTI values of gut bacterial communities from the four dietary fiber groups in jejunal **(A,C)** and cecal **(B,D)** contents.

Based on the PERMDISP results in both jejunum and cecum, the Bray-Curtis distances, but not the Jaccard distances, were significantly distinct from the null random expectation (Table 2). In both jejunum and cecum, the NTI value was more than 0 (Supplementary Figure 3A), indicating that the bacterial communities clustered phylogenetically. Although dietary fiber altered the NTI values of bacterial communities from the jejunal and cecal contents, the NTI values in all groups were more than 0 (Figures 6A,B). In the jejunum and cecum bacterial communities, the majority of the βNTI values were between −2 and + 2 (Supplementary Figure 3B), indicating they were dominated by stochastic processes. Consistent with the NTI, in both jejunal and cecal contents, dietary fiber also altered the βNTI values of bacterial communities, however, most of these values were still between −2 and + 2 (Figures 6C,D).

**TABLE 2:** Bray-Curtis and Jaccard significance tests of centroid differences between the observed communities and the null model simulations for the four dietary groups in the cecum and jejunum.

		Bray-Curtis				Jaccard			
		Actual centroid	Null centroid	*F*	*P*	Actual centroid	Null centroid	*F*	*P*
Gut region	Jejunum	0.345	0.075	130.410	**<0.001**	0.346	0.354	0.267	0.608
	Cecum	0.233	0.048	252.660	**<0.001**	0.154	0.154	<0.001	0.985
Jejunum	Control	0.334	0.078	16.170	**0.002**	0.323	0.355	0.354	0.565
	Group I	0.289	0.043	174.660	**<0.001**	0.300	0.298	0.005	0.946
	Group II	0.296	0.087	25.749	**<0.001**	0.302	0.307	0.091	0.770
	Group III	0.292	0.062	38.963	**<0.001**	0.264	0.251	0.168	0.691
Cecum	Control	0.202	0.048	64.883	**<0.001**	0.150	0.150	0.001	0.974
	Group I	0.181	0.038	32.540	**<0.001**	0.135	0.135	0.001	0.968
	Group II	0.195	0.049	113.030	**<0.001**	0.133	0.133	0.002	0.965
	Group III	0.189	0.041	63.992	**<0.001**	0.125	0.125	<0.001	0.987

*A permutational analysis of multivariate dispersions (PERMDISP) was conducted. Significant P values < 0.05 (in bold).*

We quantified the relative contributions of the five ecological processes in the four dietary fiber groups (Supplementary Figure 4). In the control group of jejunum, variable and homogeneous selection, homogenizing dispersal, and drift, were equally important to the bacterial community assembly processes (Supplementary Figure 4A), whereas in the three treatment groups of the jejunum, drift dominated the processes (Supplementary Figure 4A). In contrast to the jejunum, in all four dietary fiber groups of the cecum, drift dominated the community assembly processes (Supplementary Figure 4B).

**FIGURE 7:**
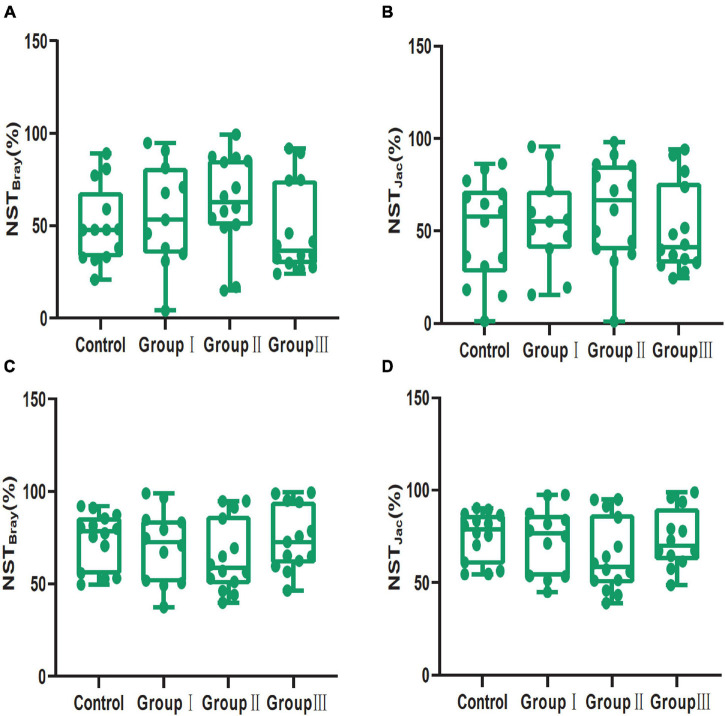
Community assembly process measurements using NST indices based on Bray-Curtis **(A,C)** and Jaccard **(B,D)** distances for the four dietary fiber groups in the jejunal **(A,B)** and cecal **(C,D)** contents.

The average value of NST based on the Bray-Curtis and Jaccard distances were 54.10 and 54.16% in the jejunum, while they were 70.82 and 71.42% in the cecum, indicating that stochastic processes were over deterministic processes in structuring gut bacterial communities in the jejunum and cecum, and the stochastic processes played a more important role in structuring bacterial community in the cecum than in the jejunum (Mann-Whitney U test: Bray-Curtis distance, *P* < 0.001; Jaccard distances, *P* < 0.001) (Supplementary Figure 5). The NST based on the Bray-Curtis distance index showed that in the jejunum, deterministic and stochastic processes were equally important to control the bacterial community assembly of the control group (50.27%), whereas stochastic processes dominated the community assemblies of group I (55.68%) and group II (63.01%), and deterministic processes dominated those in group III (47.52%) (Figure 7A). The NST based on Jaccard distances also confirmed that deterministic and stochastic processes were equally important to the assembly processes of the control group (50.12%) and group III (50.82%), while stochastic processes primarily controlled group I (54.81%) and group II (61.04%) (Figure 7B). In contrast to the jejunum, the NST based on Bray-Curtis and Jaccard distance indices showed that all groups in the cecum were mainly governed by stochastic processes (Bray-Curtis: 73.73, 70.35, 64.84, and 74.56%; and Jaccard: 74.93, 72.98, 64.56 and 73.76%; for the control group, group I, group II, and group III respectively) (Figures 7C,D).

## Discussion

In this study, we found that the composition and structure of bacterial communities in the cecum and jejunum of Durco × Bamei crossbred pigs displayed spatial heterogeneity (Figures 1–3). This is consistent with the findings in commercial pigs and cattle whose dominant bacterial species are different between small and large intestine, and commercial pigs possessed higher species richness in large intestine than in the small intestine, while cattle possess more species in small intestine than in the large intestine (Mao et al., 2015; Quan et al., 2018). The spatial heterogeneity of the bacterial community can be attributed to the variation in the structure and function of the gastrointestinal tracts at different regions, resulting in differences in pH, secretions, and gut motility (Salonen and de Vos, 2014; Mao et al., 2015; Zhang et al., 2018). A previous study on Duroc × Landrace × Large White pigs showed that the luminal pH values were distinct among different gut regions (Zhang et al., 2018). The epithelial cells in the small intestine are capable of secreting several kinds of enzymes, such as protease, aminopeptidase, and sucrase (Brunsgaard, 1997). Furthermore, bile and pancreatic juice from the liver and pancreas are also transported into the jejunum (Borja et al., 1964). However, the main secretion of the epithelial cells in the cecum is mucus, which has limited food digestive functions (Brunsgaard, 1997). Altogether, the differences in the function and secretion of epithelial cells in the jejunum and cecum create a distinct microenvironment in the two gut regions (Brunsgaard, 1997; Salonen and de Vos, 2014), resulting in differences in the composition and structure of the bacterial community.

Dietary fiber is an important factor that has been proved in many mammals to alter the composition and structure of the host gut bacterial community (David et al., 2014; Morrison et al., 2020), including in different breeds of pigs (Jha and Leterme, 2012; Heinritz et al., 2016; Liu et al., 2018; Tao et al., 2019; Pu et al., 2020). In agreement with these results, our results also showed that dietary fiber was a significant factor in shaping gut bacteria in Duroc × Bamei crossbred pigs (Figures 1, 2, 4 and Table 1). There are two plausible interpretations to explain the important role of dietary fiber in shaping the gut communities of our experimental pigs. Firstly, most of dietary fiber is carbohydrate which is the primary source of carbon and energy for gut bacteria (Sonnenburg et al., 2016). Many bacterial genera, such as *Lactobacillus* and *Bifidobacterium*, ferment dietary fiber to produce short-chain fatty acid (Zhao et al., 2018). Thereby, adding the dietary fiber into the chow increased the reproduction rates of well adapted bacterial taxa that can utilize dietary fiber, resulting in the variation in composition and structure of the gut bacteria in our pigs. Our results confirm this explanation, as the relative abundance of bacterial phyla (such as *Spirochaetae* and *Fibrobacteres*) and bacterial genera (such as *Turicibacter* and unidentified *Prevotellaceae*) which are able to degrade dietary fibers (Mao et al., 2012; Le Sciellour et al., 2018; Calusinska et al., 2020; Liu et al., 2021) increased either in jejunum or cecum with the increase in dietary fiber (Figures 1C,D). Secondly, a high proportion of dietary fiber in chow negatively impacted the host digestibility of nutrients and energy (Urriola and Stein, 2010), and was reported to induce an increase in bacteria (e.g., *Bacteroides*), which can promote the utilization of more host polysaccharides and fat (Sonnenburg et al., 2005; Wu et al., 2011), to balance energy expenditure.

In addition, we found that dietary fiber significantly increased both the Shannon and Simpson indices of the gut microbial community in the jejunum and cecum (Figures 3A,B,D,E), and that the phylogenetic α-diversity significantly increased in group II of the cecal content samples compared with those of the control (Figure 3F). Our results are in line with the reports of several studies (David et al., 2014; Heinritz et al., 2016; Tao et al., 2019; Pu et al., 2020). For example, adding appropriate fiber in the diet significantly increases the α-diversity of the gut microbial community of finishing pigs (Pu et al., 2020). The high diversity of the gut bacteria community in the experimental pigs indicated that the pigs were healthy and capable of effectively digesting dietary fiber and other food components (Clarke et al., 2014; Tap et al., 2015). Therefore, the diversity of the bacterial community can potentially act as a predictor of host tolerance to dietary fiber. However, overly high proportions of dietary fiber in chow could reduce the community richness according to the intermediate disturbance hypothesis (Connell, 1979), and as seen in our results: the α-diversity of the bacterial community of the low dietary fiber groups (control group and group I) and high fiber group (group III) was significantly lower than that of the medium dietary fiber group (group II) in cecal content samples (Figures 3D,E). The phylogenetic α-diversity of the bacterial community in the cecal content samples was also significantly higher in group II than that in the control (Figure 3F). Similar results were also found in a study on Suhuai pigs, which showed that the pigs eating the chow with 14% dietary fiber had the highest α-diversity, while the diversity indices decreased as the proportion of dietary fiber increased (Pu et al., 2020).

Additionally, our results indicated that both the gut regions and dietary fiber significantly altered the β-diversity of the bacterial taxa in the jejunal and cecal content samples (Figures 2, 4), in line with several studies (Wu et al., 2011; David et al., 2014; Heinritz et al., 2016). As we discussed, the microenvironment differed in the different gut regions, which may have led to variations. Therefore, the gut regions could also be a potential factor in shaping the bacterial community β-diversity. Similarly, in sows, the gut bacterial community in gestation was clearly separated from that in lactation (Liu et al., 2019). This could explain why the physiological functions of individuals during different physiological processes were distinctly different (Liu et al., 2019). Dietary fiber has been reported to play an important role in shaping bacteria β-diversity in many studies (Sonnenburg et al., 2016; Tao et al., 2019; Pu et al., 2020). For example, increasing the proportion of defatted rice bran in the basal diet can potentially enhance the relative abundance of specific microbiota, resulting in β-diversity variation (Pu et al., 2020). Our results also showed that in cecal content samples, interindividual dissimilarity based on Jaccard distances significantly decreased with the increase of dietary fiber (Figure 4D). A high proportion of dietary fiber in chow was an advantage for the bacteria capable of fermenting dietary fiber (Yu et al., 2016; Zhao et al., 2018), resulting in the bacterial communities being more similar in composition and structure. Weaned piglets also showed similar results, as individuals fed with different sources of dietary fiber shared more OTUs than those in the control (Zhao et al., 2018).

Bacterial networks were generally more complex in cecal content samples than in jejunal content samples (Supplementary Figure 2). This can be explained by the fact that the cecum is the main gut region for pigs to ferment and digest food resources using bacteria (Abelilla and Stein, 2019). The proportion of positive associations among the 20 most abundant genera increased with the increase of dietary fiber in both jejunal and cecal content samples, except in group III of the jejunum (Figure 5). The high proportion of positive correlations indicated strong competition among the 20 most abundant gut bacteria (Bertness and Callaway, 1994). Our results support the stress gradient hypothesis, which predicts that positive associations will increase along with stress in ecological communities (Bertness and Callaway, 1994). Network complexities greatly increased along with dietary fiber in jejunal content samples, based on the number of edges, average weighted degrees, density, and average clustering coefficient (Wei et al., 2020; Figure 5A). This is in agreement with the metabolic theory of ecology, which predicts that the ecological networks will shift from simple to complex as individual metabolic processes become more active and the growth rate increases (Brown et al., 2004). The addition of dietary fiber to swine chow may increase the metabolic activity and growth rate of gut bacteria (Zhao et al., 2018), therefore the bacterial networks became more complex in the high dietary fiber groups. However, the network complexities of cecal content samples decreased with the increase of dietary fiber (Figure 5B), which was not supported by the theory (Brown et al., 2004). One of the potential explanations is that bacteria can interact antagonistically in a community, which is defined as amensalism, and the increase in cellulose decomposing bacteria may be detrimental to the associations of other bacteria in the gut ecosystem, resulting in a less complexity in the networks (Zengler and Zaramela, 2018).

The results of an abundance-weighted null model test (PERMDISP) based on Bray-Curtis dissimilarity matrices showed that deterministic processes control the gut bacteria assemblies of all of the samples (Table 2). In contrast, the results of the test based on Jaccard dissimilarity matrices showed that the bacterial community primarily assembled stochastically rather than deterministically (Table 2). Additionally, the NTI and βNTI did not differ significantly from a null distribution in any of the samples (Figure 6), indicating that stochastic processes dominated the community assembly processes, according to the framework of Stegen et al. (2012). Using phylogenetic information (NTI and βNTI) to infer the ecological processes should assume that the traits regulating assembly processes are phylogenetically conserved (Stegen et al., 2012). The contradictory results may be explained by the fact that the traits regulating assembly processes are not phylogenetically conserved in our result (Supplementary Figure 6). Therefore, we applied the normalized stochasticity ratio (NST) to help confirm the bacterial community assembly processes, which does not require any additional premises (Ning et al., 2019). Although the NST has been developed recently, it has been applied widely (Ning et al., 2019; Le Moigne et al., 2020; Li P. et al., 2020). Compared with previous approaches, which were used to infer the importance of deterministic and stochastic processes in determining ecological communities, the NST has many advantages, including high accuracy and precision (Ning et al., 2019). Additionally, the NST could quantify the relative importance of stochastic processes in determining ecological communities, which cannot be achieved with the other approaches (Ning et al., 2019).

The NST index suggested that stochastic processes and deterministic processes were equally important in structuring bacterial communities from jejunum content samples, while the stochastic processes were over deterministic processes in structuring bacterial communities in cecum (Supplementary Figure 5). Previous studies showed that host development stages could influence the selection pressure, resulting in variation of bacterial community assembly processes, which can attribute to the distinct functions of host gut in different development stages (Burns et al., 2016; Yan et al., 2016; Holt et al., 2020). Our results indicate that gut region also is an important factor in shaping assembly processes of gut bacterial communities for their distinct functions. Additionally, stochastic processes dominated the structure of cecal bacterial community which can account for the great variation of cecal bacterial communities than that in the jejunum at corresponding dietary fiber levels (Figure 3). High dietary fiber chow shifted the gut bacterial community assembly from stochastic processes to deterministic processes in the jejunum (Figures 7A,B). Although this variation was not statistical significance, it was meaningful change according the definition of NST. The decrease of nutrition could trigger competitive exclusion which can shift bacterial community from stochastic processes to deterministic processes (Pontarp and Petchey, 2016; Ning et al., 2019). For example, increasing the organic carbon input to the groundwater microbial community shifted the community assembly from deterministic to stochastic processes, and the assembly processes turned to deterministic with the nutrition consumption (Ning et al., 2019); deterministic processes dominated the microbial community assembly in the oligotrophic ocean (Allen et al., 2020). Therefore, the addition of dietary fiber to the Duroc × Bamei crossbred pigs’ chow may have reduced the nutrition level and led to deterministic processes dominating the bacterial community assembly of the high dietary fiber group in the jejunum (Figures 7A,B). That stochastic processes dominated the bacterial community assembly in all groups of the cecum may be attributed to the fact that many bacterial taxa in the cecum are able to ferment and digest dietary fiber (Abelilla and Stein, 2019) and can tolerate the low nutrition of the food resources.

## Conclusion

In conclusion, the bacterial community dynamics between the jejunum and cecum showed a distinct spatial heterogeneity across different graded levels of dietary fiber in Durco × Bamei crossbred pigs. Dietary fiber increased the complexity of the bacterial network in jejunal content samples, but reduced it in cecal content samples. Meanwhile, deterministic processes dominated the bacterial community of jejunal content samples in the high dietary fiber group, while the percentage of the deterministic and stochastic processes was very close in low and medium dietary fiber groups. However, the stochastic processes played a greater role in structuring the bacterial communities of all the cecal content samples. Altogether, these findings expand our knowledge of the ecological processes that govern microbial community assembly in livestock gut ecosystems in response to dietary fiber intake.

## Data Availability Statement

The 16S rDNA and the shotgun metagenomic data in this study can be freely retrieved from the NCBI Sequence Read Archive with Project Accession No. PRJNA685300. Other data can be found in the article/Supplementary Material.

## Ethics Statement

The animal study was reviewed and approved by the Department of Animal Ethics Committee of QingHai University (Approval number: NQH2019102). Written informed consent was obtained from the owners for the participation of their animals in this study.

## Author Contributions

YZ and GW designed the study. LZ, LW, GW, and WS performed research and collated the samples. YZ, LZ, SJ, CF, XT, HF, and SR carried out the data analyses. XT produced the initial draft of the manuscript. YZ, SJ, GW, LZ, and CF contributed to the revision of the manuscript. All authors contributed to the article and approved the submitted version.

## Conflict of Interest

The authors declare that the research was conducted in the absence of any commercial or financial relationships that could be construed as a potential conflict of interest.

## Publisher’s Note

All claims expressed in this article are solely those of the authors and do not necessarily represent those of their affiliated organizations, or those of the publisher, the editors and the reviewers. Any product that may be evaluated in this article, or claim that may be made by its manufacturer, is not guaranteed or endorsed by the publisher.

## Footnotes

1
http://qiime.org/scripts/split_libraries_fastq.html

2
https://github.com/torognes/vsearch/

3
http://www.drive5.com/uparse/

4
http://www.arb-silva.de/

5
https://www.r-project.org/

## Supplementary Material

Supplementary Figure 1 The relative abundance of the 15 most abundant bacterial phyla **(A)** and 20 most abundant bacterial genera **(B)** of jejunal and cecal content samples. Comparisons between the two gut regions for the differences in α-diversity (Shannon’s index, Simpson’s index, and Phylogenetic index for **(C–E)**, respectively). Differences were assessed by the Wilcoxon test and are denoted as follows: **P* < 0.05; ***P* < 0.01; ****P* < 0.001.

Supplementary Figure 2 Co-occurrence networks of the 20 most abundant genera in jejunal and cecal content samples. Each line (blue: negative; red: positive) represents a Spearman’s correlation coefficient greater than + 0.5 or lower than −0.5.

Supplementary Figure 3 Box plot of NTI and βNTI values of gut bacterial communities from jejunal **(A)** and cecal **(B)** content samples.

Supplementary Figure 4 Summary of the contribution of the ecological processes that determined community assembly of gut microbiota from the four dietary fiber groups in jejunal **(A)** and cecal **(B)** contents.

Supplementary Figure 5 Comparisons between the two gut regions for the differences in Community assembly process measurements using NST indices based on Bray-Curtis **(A)** and Jaccard **(B)** distances.

Supplementary Figure 6 Mantel correlation between the pairwise matrix of OTU niche distances and the phylogenetic distances in gut microbiota of Durco × Bamei pigs with 999 permutations. Significant correlations (*P* < 0.05) of phylogenetic signals in species ecological niches are labeled as solid circles, whereas non-significant correlations are labeled as hollow circles.

Supplementary Table 1 Ingredient composition, nutrient, and energy content of the diet.

Supplementary Table 2 Nutrient and energy content of the silage.

Supplementary Table 3 Two-way ANOVA showing the effects of gut region and dietary fiber on the α-diversity of the bacterial communities.
