# Supplementary figures and images for: Dietary Fiber Influences Bacterial Community Assembly Processes in the Gut Microbiota of Durco × Bamei Crossbred Pig
Source: Front Microbiol. 2021 Dec 8;12:688554. doi: 10.3389/fmicb.2021.688554 (PMC8693415; doi:10.3389/fmicb.2021.688554)

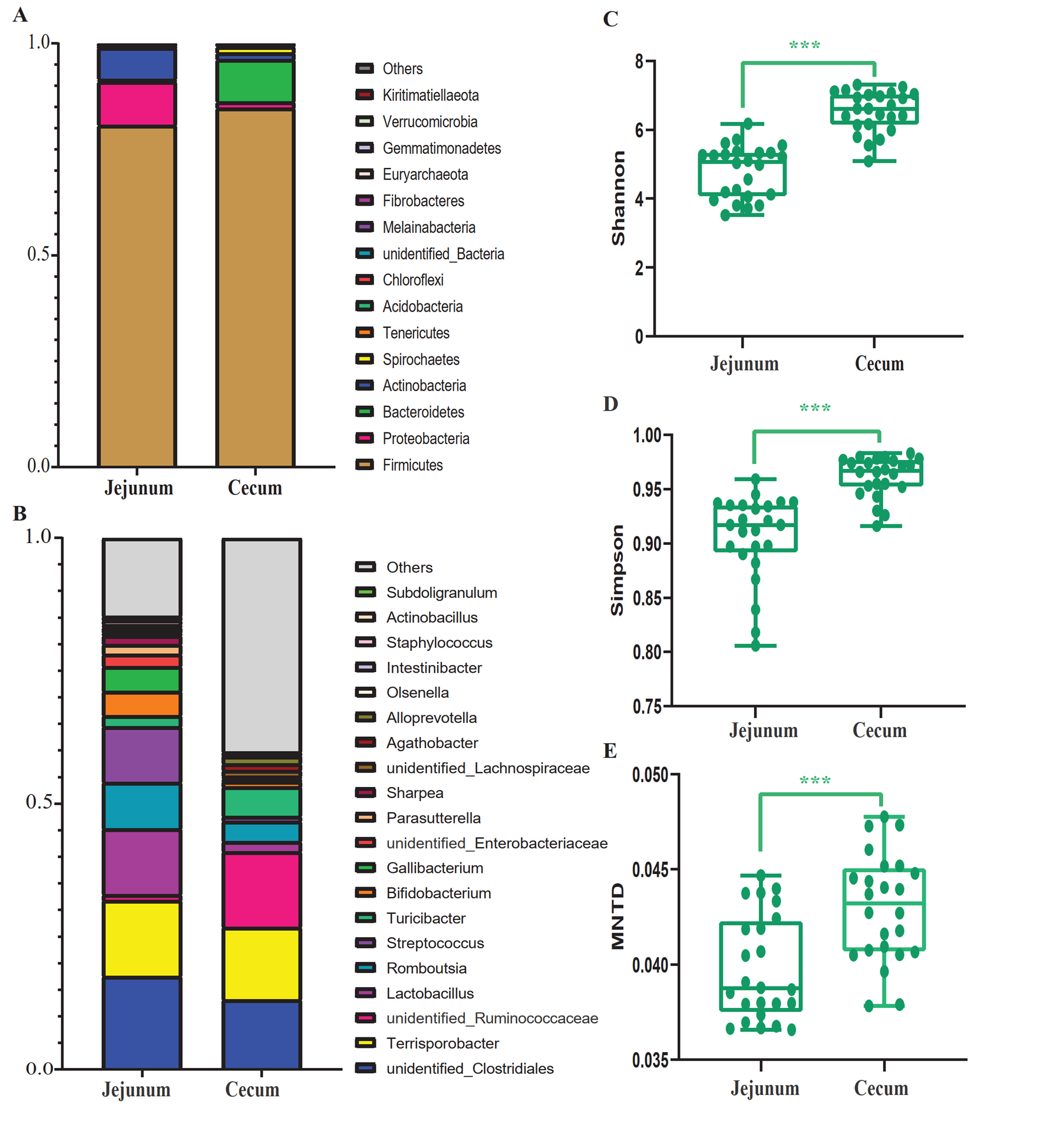

Supplement: Supplementary Figure 1 — The relative abundance of the 15 most abundant bacterial phyla (A) and 20 most abundant bacterial genera (B) of jejunal and cecal content samples. Comparisons between the two gut regions for the differences in α-diversity (Shannon’s index, Simpson’s index, and Phylogenetic index for (C–E), respectively). Differences were assessed by the Wilcoxon test and are denoted as follows: *P < 0.05; **P < 0.01; ***P < 0.001. [file Image_1.TIF]

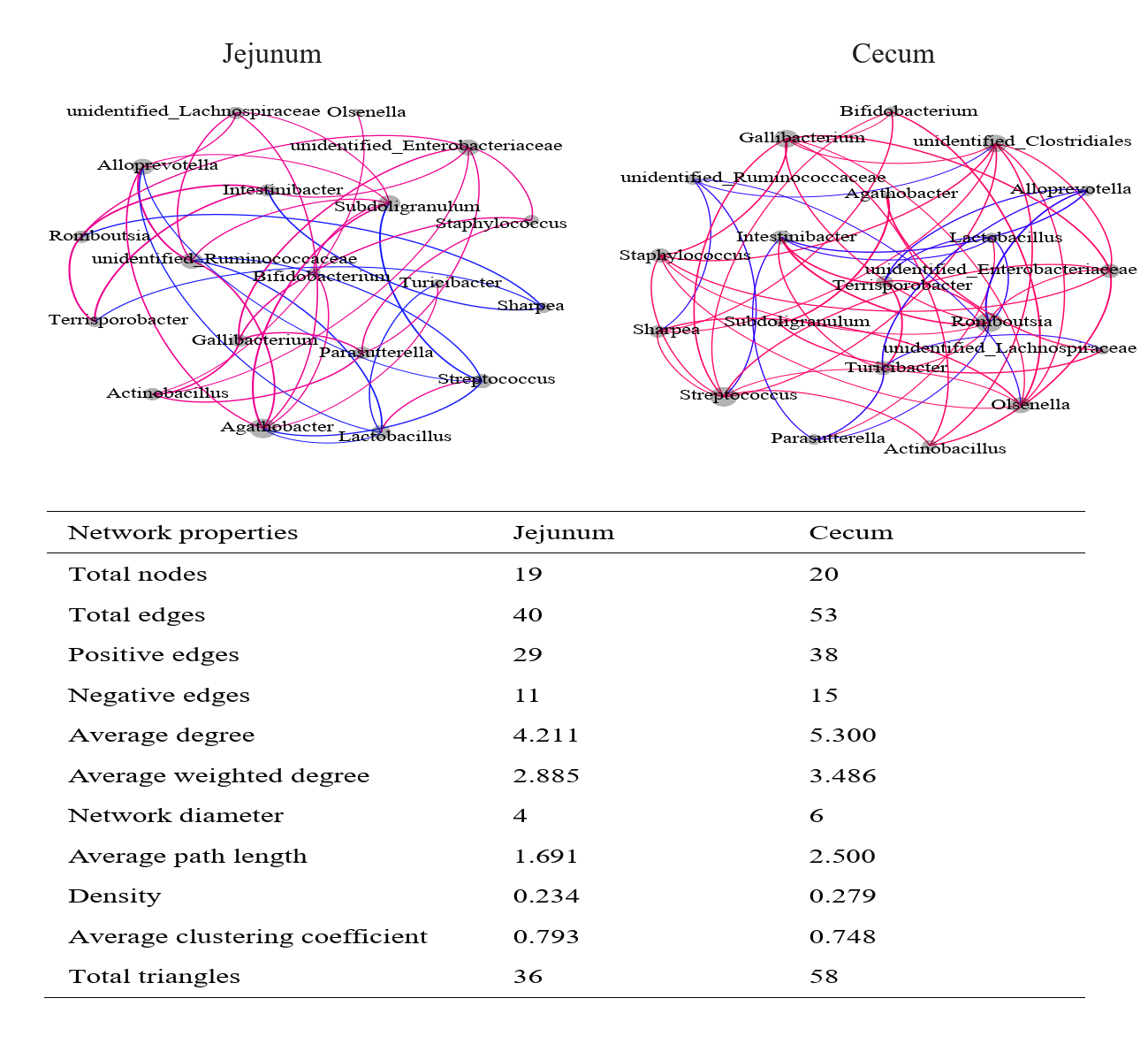

Supplement: Supplementary Figure 2 — Co-occurrence networks of the 20 most abundant genera in jejunal and cecal content samples. Each line (blue: negative; red: positive) represents a Spearman’s correlation coefficient greater than + 0.5 or lower than −0.5. [file Image_2.TIF]

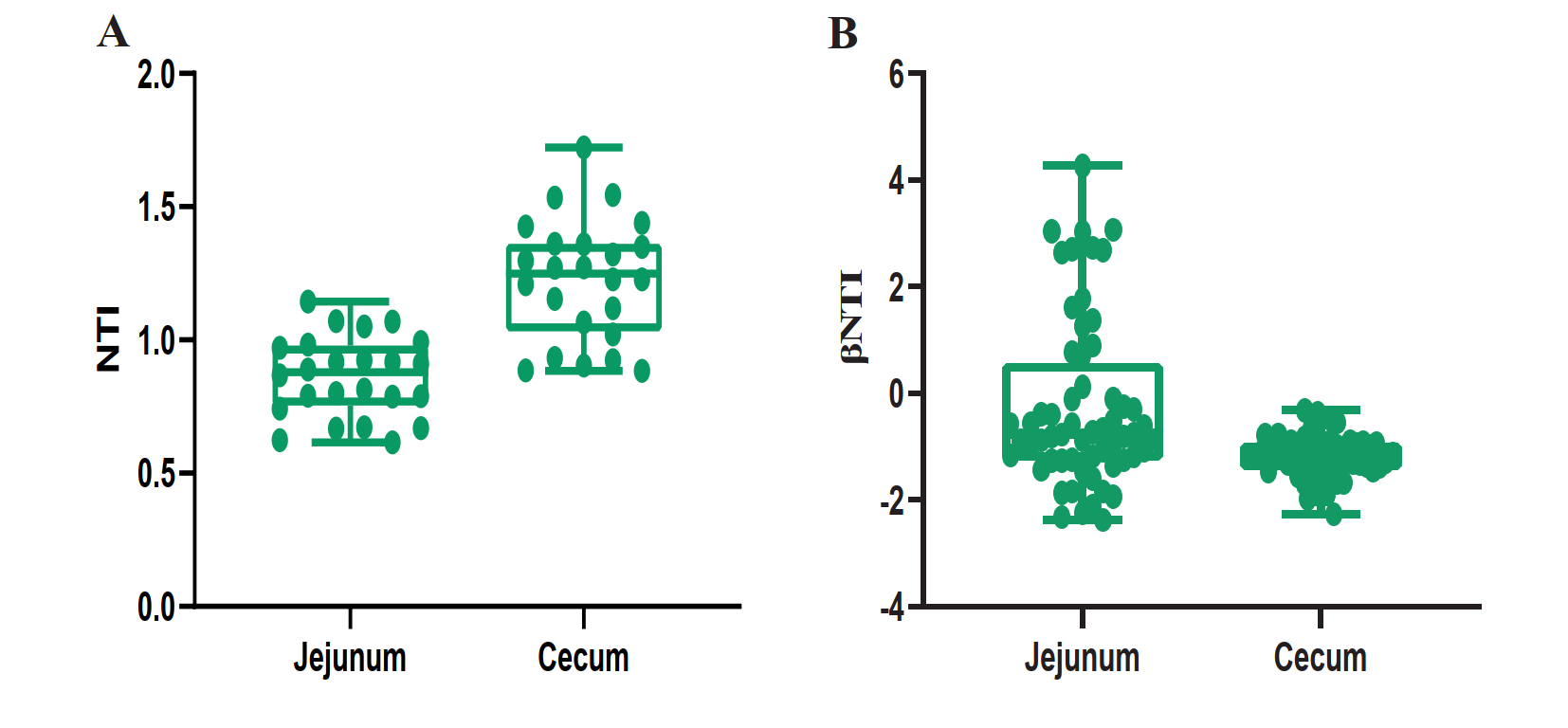

Supplement: Supplementary Figure 3 — Box plot of NTI and βNTI values of gut bacterial communities from jejunal (A) and cecal (B) content samples. [file Image_3.TIF]

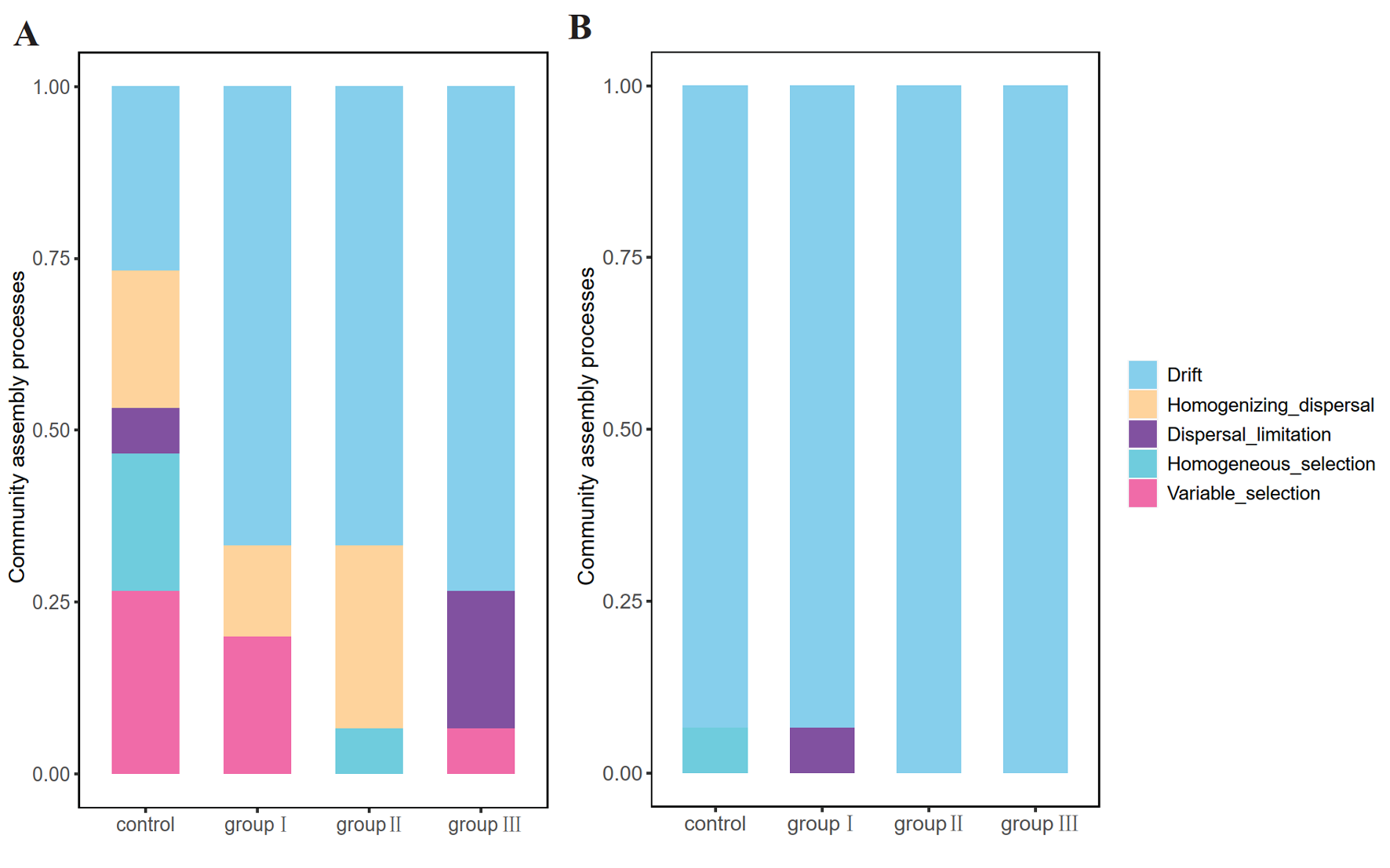

Supplement: Supplementary Figure 4 — Summary of the contribution of the ecological processes that determined community assembly of gut microbiota from the four dietary fiber groups in jejunal (A) and cecal (B) contents. [file Image_4.TIF]

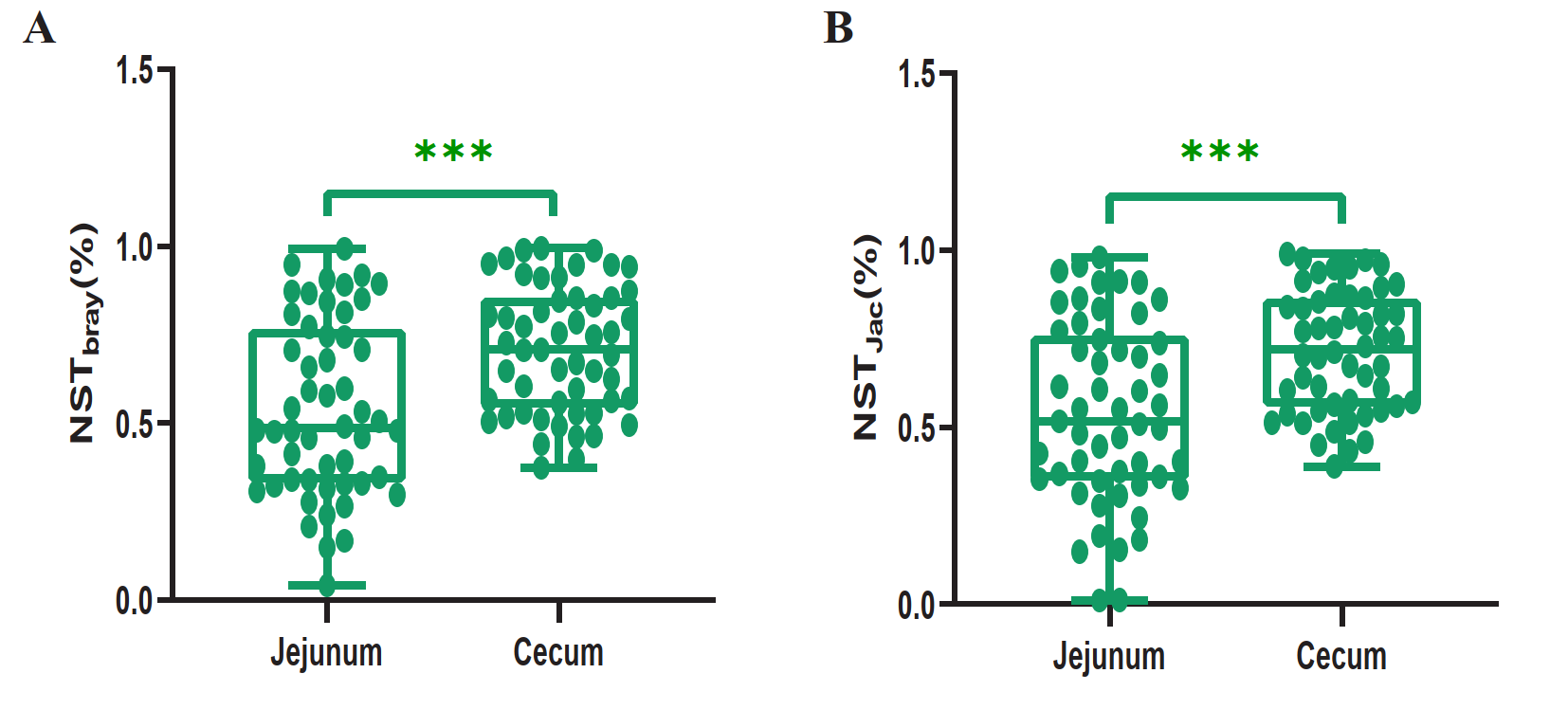

Supplement: Supplementary Figure 5 — Comparisons between the two gut regions for the differences in Community assembly process measurements using NST indices based on Bray-Curtis (A) and Jaccard (B) distances. [file Image_5.TIF]

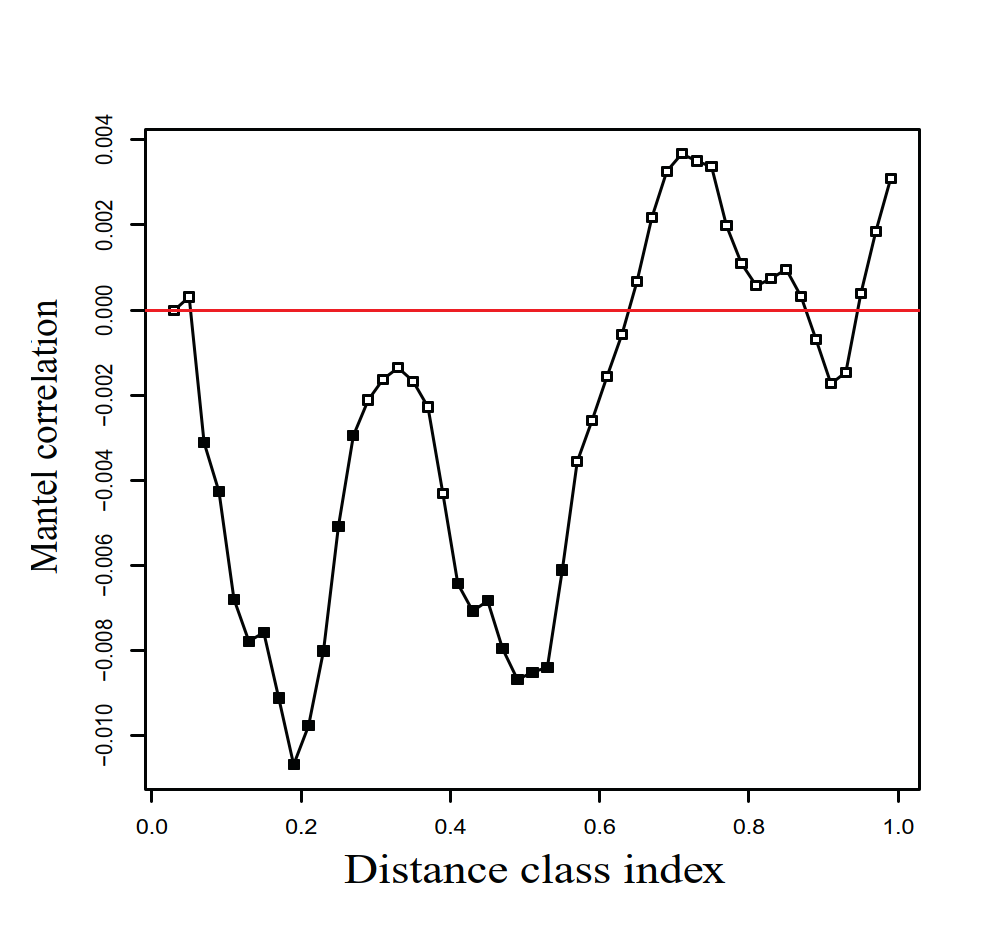

Supplement: Supplementary Figure 6 — Mantel correlation between the pairwise matrix of OTU niche distances and the phylogenetic distances in gut microbiota of Durco × Bamei pigs with 999 permutations. Significant correlations (P < 0.05) of phylogenetic signals in species ecological niches are labeled as solid circles, whereas non-significant correlations are labeled as hollow circles. [file Image_6.TIF]
