# Supplementary material for: Dietary Fiber Influences Bacterial Community Assembly Processes in the Gut Microbiota of Durco × Bamei Crossbred Pig
Source: Front Microbiol. 2021 Dec 8;12:688554. doi: 10.3389/fmicb.2021.688554 (PMC8693415; doi:10.3389/fmicb.2021.688554)
Supplement: Supplementary Table 1 — Ingredient composition, nutrient, and energy content of the diet. [file Table_1.DOCX]

Table S1. Ingredient composition, nutrient and energy content of the diet.

| Ingredient (%) | Control group | GroupⅠ | GroupⅡ | GroupⅢ |
| --- | --- | --- | --- | --- |
| **Basic dietary** |  |  |  |  |
| Corn | 79.13 | 70.57 | 62.38 | 53.96 |
| Soybean meal | 13.17 | 12.41 | 12.47 | 12.58 |
| Rapeseed meal | 4.00 | 4.00 | 4.00 | 0.00 |
| Broad bean straw | 0.00 | 10.00 | 17.00 | 24.00 |
| **silage** |  |  |  |  |
| Soybean oil | 0.00 | 0.00 | 1.42 | 2.90 |
| Dicalcium phosphate | 1.64 | 1.07 | 1.13 | 1.18 |
| Stone powder | 1.06 | 0.95 | 0.60 | 0.37 |
| Premix | 1.00 | 1.00 | 1.00 | 1.00 |
| **Nutrition level** |  |  |  |  |
| Metabolic energy（KC/Kg) | 2988 | 2890 | 2890 | 2890 |
| Crude protein（%） | 14.10 | 14.00 | 14.00 | 14.00 |
| Crude fiber（%） | 2.40 | 4.20 | 5.50 | 6.80 |
| Ca（%） | 0.80 | 0.74 | 0.70 | 0.70 |
| P（%） | 0.50 | 0.50 | 0.50 | 0.50 |

The premix provided per kilogram of diets: V_A_ 13000 IU, V_D_ 33000 IU, V_E_ 35 mg, VK 33 mg, V_B_ 12.5 mg, V_B_26 mg, V_B_ 63 mg, V_B_ 120.25 mg, Nicotinicacid 25 mg, Pantothenicacid 15 mg, Biotin 0.15 mg, Cu 150 mg, Fe 80 mg, Zn 80 mg, Mn 10 mg, I 0.3 mg, Se 0.2 mg.
