# Supplementary material for: Dietary Fiber Influences Bacterial Community Assembly Processes in the Gut Microbiota of Durco × Bamei Crossbred Pig
Source: Front Microbiol. 2021 Dec 8;12:688554. doi: 10.3389/fmicb.2021.688554 (PMC8693415; doi:10.3389/fmicb.2021.688554)
Supplement: Supplementary Table 2 — Nutrient and energy content of the silage. [file Table_2.DOCX]

Table S2. Nutrient and energy content of the silage.

| Ingredient |  |
| --- | --- |
| Dry matter (g/kg) | 95 |
| Starch (g/kg) | 11 |
| Energy (KJ/kg) | 1890 |
| Crude protein (%) | 1.21 |
| Neutral detergent fiber (%) | 6.4 |
| Acid detergent fiber (%) | 5.2 |
| Ammoniacal nitrogen (mg/kg) | 380.74 |
| Soluble sugar (g/kg) | 0.65 |
