# Supplementary material for: Dietary Fiber Influences Bacterial Community Assembly Processes in the Gut Microbiota of Durco × Bamei Crossbred Pig
Source: Front Microbiol. 2021 Dec 8;12:688554. doi: 10.3389/fmicb.2021.688554 (PMC8693415; doi:10.3389/fmicb.2021.688554)
Supplement: Supplementary Table 3 — Two-way ANOVA showing the effects of gut region and dietary fiber on the α-diversity of the bacterial communities. [file Table_3.docx]

Table S3. Two-way ANOVA showing the effects of gut region and dietary fiber on the α-diversity of gut bacterial communities.

|  | Sums of squares | degree of freedom | Mean squares | *F* | *P* |
| --- | --- | --- | --- | --- | --- |
| Region |  |  |  |  |  |
| Shannon | 35.892 | 1 | 35.892 | 111.134 | **.000** |
| Simpson | .037 | 1 | .037 | 47.621 | **.000** |
| Phylogenetic | .000 | 1 | .000 | 18.527 | **.000** |
|  |  |  |  |  |  |
| Fiber |  |  |  |  |  |
| Shannon | 5.923 | 3 | 1.974 | 6.113 | **.002** |
| Simpson | .008 | 3 | .003 | 3.533 | **.023** |
| Phylogenetic | < 0.001 | 3 | < 0.001 | 3.322 | **.029** |
|  |  |  |  |  |  |
| Region & Fiber |  |  |  |  |  |
| Shannon | 2.281 | 3 | .760 | 2.354 | .086 |
| Simpson | .004 | 3 | .001 | 1.904 | .144 |
| Phylogenetic | < 0.001 | 3 | < 0.001 | 1.174 | .332 |

*P* values < 0.05 in bold.
